# Medicaid Expansion and Perinatal Health Outcomes: A Quasi-Experimental Study

**DOI:** 10.1007/s10995-023-03879-y

**Published:** 2024-01-20

**Authors:** Sepideh Modrek, Daniel F. Collin, Rita Hamad, Justin S. White

**Affiliations:** 1 Health Equity Institute, San Francisco State University, San Francisco, CA USA; 2 Department of Social and Behavioral Sciences, Harvard School of Public Health, Boston, MA USA; 3 Philip R. Lee Institute for Health Policy Studies, University of California San Francisco, San Francisco, CA USA; 4 Department of Health Law, Policy and Management, Boston University School of Public Health, 715 Albany Street, Boston, MA 02118 USA

**Keywords:** Medicaid Expansion, Birthing Parent Health, Infant Health, PRAMS

## Abstract

**Objective:**

There has been little evidence of the impact of preventive services during pregnancy covered under the Affordable Care Act (ACA) on birthing parent and infant outcomes. To address this gap, this study examines the association between Medicaid expansion under the ACA and birthing parent and infant outcomes of low-income pregnant people.

**Methods:**

This study used individual-level data from the 2004–2017 annual waves of the Pregnancy Risk Assessment Monitoring System (PRAMS). PRAMS is a surveillance project of the Centers for Disease Control and Prevention and health departments that annually includes a representative sample of 1,300 to 3,400 births per state, selected from birth certificates. Birthing parents’ outcomes of interest included timing of prenatal care, gestational diabetes, hypertensive disorders of pregnancy, cigarette smoking during pregnancy, and postpartum care. Infant outcomes included initiation and duration of breastfeeding, preterm birth, and birth weight. The association between ACA Medicaid expansion and the birthing parent and infant outcomes were examined using difference-in-differences estimation.

**Results:**

There was no association between Medicaid expansion and the outcomes examined after correcting for multiple testing. This finding was robust to several sensitivity analyses.

**Conclusions for Practice:**

Study findings suggest that expanded access to more complete insurance benefits with limited cost-sharing for pregnant people, a group that already had high rates of insurance coverage, did not impact the birthing parents’ and infant health outcomes examined.

**Supplementary Information:**

The online version contains supplementary material available at 10.1007/s10995-023-03879-y.

## Significance

Pregnant people had high rates of insurance coverage before the Affordable Care Act, but insurance instability remains high during the perinatal period. The ACA made a substantial number of changes to the cost-sharing of preventive benefits for pregnant people. There is limited research on changes in outcomes related to new preventive benefits for pregnant people.

We examined several perinatal outcomes for low-income pregnant people after full enactment of ACA provisions. We find little change in the outcomes examined. Results suggest that ACA policies and insurance expansion that predate the ACA were necessary but insufficient to improve these more intractable outcomes.

**Supplementary Information:**

The online version contains supplementary material available at 10.1007/s10995-023-03879-y.

## Introduction

Overall, insurance coverage, access, and affordability of healthcare have improved in the US since the final enactment of the Affordable Care Act (ACA) in 2014 (Lee et al., 2020). Yet, even before the ACA, pregnant people were one of the few groups that had consistent access to insurance, especially through categorical eligibility for Medicaid among low-income pregnant people. In 2009, 97% of pregnant people had health insurance coverage for prenatal care, and 99% reported having had health insurance coverage at some point during their pregnancy (D’Angelo et al., 2015). Despite these high coverage rates, studies have documented high rates of insurance instability during the perinatal period (Johnston et al., 2021). In 2009 and 2017 roughly 30% of pregnant people changed insurance coverage during pregnancy (Johnston et al., 2021). High rates of insurance instability during the perinatal period may impact the quantity and quality of healthcare utilization and subsequent health outcomes.

A central provision of the ACA expanded Medicaid to low-income adults. A recent review highlights that many low-income pregnant people in Medicaid-expanding states gained Medicaid coverage prior to their pregnancy, thus increasing the number of pregnancies with continuous insurance coverage (i.e., having the same coverage before, during, and after pregnancy) and decreasing insurance churn during and after pregnancy (i.e., moving between insurance and uninsurance or switching insurers) (Bellerose et al., 2022). A complementary ACA provision mandated that all private and government health insurers, including Medicaid, cover and eliminate cost-sharing for essential benefits during pregnancy. The essential benefits provision for pregnant people included tobacco cessation counseling and interventions; preeclampsia prevention; breastfeeding support, counseling, and equipment rental; and gestational diabetes screening (Lee et al., 2020). Thus, in states that expanded Medicaid, pregnant people experienced improved and earlier access to pre-pregnancy insurance, perhaps allowing them more time to access services, coordinate care, and build better relationships with their providers to take advantage of the more comprehensive coverage of preventive services during pregnancy (Dehlendorf et al., 2016; O’Malley et al., 2004).

Early, consistent, and continuous access to comprehensive insurance coverage of pregnancy-related services may improve the health of both the pregnant person and child during pregnancy, at birth, and in early infancy, especially for Medicaid-eligible and low-income people who may need more support during pregnancy. Economically disadvantaged pregnant people have higher rates of smoking, preeclampsia, and diabetes, and lower rates of prenatal visits and breastfeeding initiation (Anstey et al., 2017; Ross et al., 2019; Tong et al., 2009). Some face higher barriers to accessing care due in part to language and immigration status (Admon et al., 2021; Liou, 2018).

Prior studies have focused on the impact of the Medicaid expansion provision on insurance status and birthing parent and child outcomes. Several studies have found that the Medicaid expansion was associated with increased prenatal insurance and continuous Medicaid coverage but mixed findings on changes overall in insurance rates (Bellerose et al., 2022; Daw et al., 2020). Other studies have documented some improvement in birthing parents’ mental health (Margerison et al., 2021), but not with early prenatal care visits, preterm birth rates, or rates of low birth weight (Bellerose et al., 2022; Clapp et al., 2019). One study found improvements in birth weight outcomes for Black infants compared with White infants in expansion states (Brown et al., 2019). Another found no reduction in infant mortality rates overall but a decline among Hispanic infants (Wiggins et al., 2020).

This study adds to the existing literature by leveraging a large national data set and a quasi-experimental design to examine whether the confluence of ACA provisions—Medicaid expansion and essential benefits provision—affected healthcare utilization and health outcomes for pregnant people and their infants. In addition to pre- and post-natal care utilization, we focus on health outcomes related specifically to the newly covered preventive services under the essential benefits provision. For birthing parents, we examine timing of prenatal care, use of postpartum care, gestational diabetes, hypertensive disorders of pregnancy, and cigarette smoking during pregnancy. For infants, we examine associated outcomes including initiation and duration of breastfeeding, preterm birth, and birth weight. We hypothesize that the essential benefits provision combined with Medicaid expansion led to continuous and consistent access to physician advice and care and thus more consistent use of newly covered preventive services, especially for low-income people who have higher prenatal risks.

## Methods

### Data

Data for this study came from the 2004–2017 annual waves of the Pregnancy Risk Assessment Monitoring System (PRAMS), a surveillance project of the Centers for Disease Control and Prevention and state and local health departments. PRAMS participants include a representative sample of 1,300 to 3,400 births per year from birth certificates in each jurisdiction (i.e., state or territory) and collects survey responses on demographics and health outcomes before, during, and shortly after pregnancy. PRAMS participants represent approximately 81% of all US live births; however, PRAMS data are only released each year for sites that meet a minimum response rate threshold, ranging from 55 to 70% during the study period, and several states are omitted from the sample. The PRAMS methodology has been described elsewhere (Shulman et al., 2018).

### Sample Selection

**Fig. 1:**
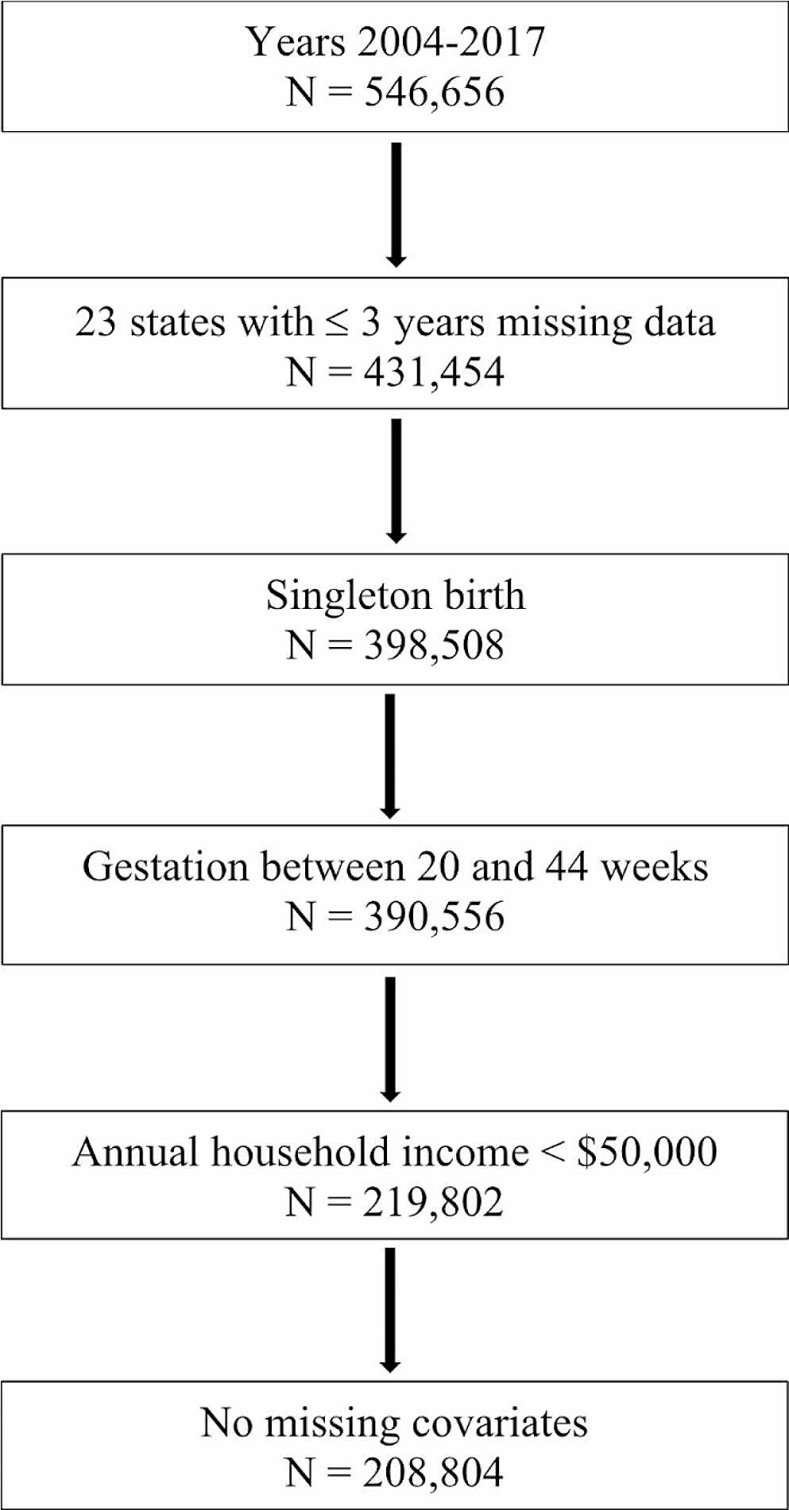
Sample Selection Flowchart. Pregnancy Risk Assessment Monitoring System (PRAMS) dataset includes linked birth certificate and birthing parents’ survey responses

We used annual PRAMS survey waves from 2004 to 2017 (*N* = 546,656). Data prior to 2004 were excluded due to differences in how birth certificate data were collected, and 2017 was the most recent year of data available at the start of our analyses. We included data from states with no more than 3 missing waves, resulting in 23 states (Fig. 1). Sixteen states had expanded Medicaid during the 2004–2017 period (AK, AR, CO, DE, HI, IL, MA, MD, MI, NJ, NY, OR, PA, RI, WA, WV) and 7 had not (ME, NE, OK, UT, WI, WY; see Supplemental Table S1). We included live-born singleton births with a gestational age of 20 to 44 weeks at delivery. We include births in households with income below $50,000 (*N* = 219,802). Birthing parents in these households are more likely to be eligible for Medicaid and affected by Medicaid eligibility and benefits requirements. We use this threshold because Medicaid income eligibility for pregnant people varied substantially over time and by state. For example, in 2011, before the implementation of the ACA, Wisconsin covered pregnant people up to 300% of the federal poverty line (FPL), which for a household of two would have been $45,000 (See Supplemental Table S2 for Medicaid income eligibility for pregnant people for included states). Moreover, “low-income” is sometimes considered 150% of the federal poverty level. For a family of four, this would be about $45,000 in 2023, close to our eligibility threshold. We further limit the sample to cases with no missing key covariates (*N* = 208,063).

### Exposure

The primary exposure was a dichotomous variable indicating whether the estimated date of conception occurred on or after the year-quarter of the state’s Medicaid expansion (i.e., 1 = post-expansion; 0 = pre-expansion and for all non-expansion states). Thirteen states expanded Medicaid effective on January 1, 2014, and three states expanded Medicaid at a later date: Michigan (April 1, 2014), Alaska (September 1, 2015), and Pennsylvania (January 1, 2015). Several states allowed low-income adults at varying income thresholds to enroll in Medicaid or Medicaid-like insurance programs (state safety net programs) prior to 2014. These states are assigned an effective date of January 1, 2014.

### Outcomes

We selected birthing parent and infant outcomes that could be affected by new regulations on insurance coverage through the ACA and Medicaid expansion. Birthing parents’ outcomes included month of first prenatal visit, diagnosis of gestational diabetes mellitus (self-reported in survey or from linked birth certificate), diagnosis of hypertensive disorders of pregnancy (from linked birth certificate), smoking status during last three months of pregnancy (self-reported in survey), and postpartum check-up visit (self-reported in survey). Hypertensive disorders of pregnancy includes both gestational hypertension and preeclampsia, which are reported as a single measure in PRAMS (“Gestational Hypertension and Preeclampsia: ACOG Practice Bulletin, Number 222,” 2020). Stable, comprehensive insurance coverage prior to and during pregnancy might reduce stress and increase care-seeking behavior. Moreover, increased screening could identify conditions like gestational diabetes and hypertensive disorders in a timely manner and lead to improved condition management over the course of the pregnancy. The birthing parents’ outcomes encompass a range of outcomes in which the impact of insurance coverage and specific benefits was expected to be more direct and immediate (e.g., first prenatal visit) or more distal and diffuse (e.g., diagnosis of gestational diabetes or hypertensive disorder). All might be expected to influence infant outcomes such as birth weight.

Infant outcomes included self-reported survey responses to whether infant was breastfed (binary) and breastfed for ≥ 1 month (binary); and outcomes from the linked birth certificate including preterm birth (< 37 weeks’ gestation), low birth weight (< 2500 g), and very low birth weight (< 1500 g). Current breastfeeding practices were asked at the time of the post-partum survey, and we used the date of birth and survey completion to construct our measure of breastfeeding for ≥ 1 month.

### Covariates

Covariates included pregnant person’s age, race/ethnicity, education, marital status, household size, and parity. For race and ethnicity, categories included non-Hispanic White, non-Hispanic Black, Hispanic/Latina, and other. While PRAMS includes more detailed information on race and ethnicity, the latter category was created due to small subgroups and potentially unstable estimates. The analytical sample includes households with incomes under $50,000. Within this income range, we include controls for income categories (less than $10,000, $10,000-$19,999, $20,000-$29,999, $30,000–$39,999, $40,000-$49,999). We also adjusted for state-level covariates that might confound the relationship between the exposure and outcome: poverty rate, unemployment rate, gross domestic product, and Earned Income Tax Credit (EITC) enrollment rate (Kaiser Family Foundation, 2020; U.S. Bureau of Labor Statistics, 2021; U.S. Census Bureau; University of Kentucky Center for Poverty Research, 2020).

### Analysis

We first tabulated descriptive statistics for sample characteristics of all pregnant people in expansion and non-expansion states. We calculated the standardized difference for each characteristic across groups, a statistic that is not influenced by the large sample size. We then estimated the effect of Medicaid expansion on birthing parent and infant outcomes using a difference-in-differences (DiD) approach. DiD is a quasi-experimental technique well suited to examining the effect of policies while accounting for secular trends in outcomes (Basu et al., 2017; Ryan et al., 2015). Our analysis leveraged the fact that some states expanded Medicaid on or after January 1, 2014, while others did not. We therefore estimated the pre-post changes in the outcomes in the treated group (low-income people in Medicaid expansion states) while subtracting or “differencing” out the changes in the outcomes in the control group (low-income people in non-expansion states). The primary independent variable in the DiD analysis is an indicator variable for whether conception occurred in a Medicaid expansion state after implementation of the policy. As this study involved multiple treatment units (i.e., states) expanding Medicaid at different time points, we followed a generalized DiD analytic approach, including fixed effects (i.e., indicator variables) for year and state to account for secular changes and time-invariant state heterogeneity, respectively (Wing et al., 2018). We followed a standard approach used in DiD analyses to estimate multivariable linear regressions for both binary and continuous outcomes due to the differences in the interpretation of interaction terms in non-linear models (Athey & Imbens, 2006; Karaca-Mandic et al., 2012). The coefficients for binary outcomes can therefore be interpreted as a percentage-point change in risk. Standard errors are clustered by state to account for correlated outcomes among individuals from the same state (e.g., due to the same state policy environment). Regressions do not include survey or analytical weights. Notably, the appropriateness of sample weighting is diminished when the goal of analysis is estimation of treatment effects rather than producing descriptive population statistics (Miratrix et al., 2018; Solon et al., 2015).

#### DiD Assumptions

One important assumption of the DiD approach is that pre-post differences in outcomes of interest would be the same in the treated and control groups in the absence of the Medicaid expansion. While this counterfactual fundamentally cannot be tested, we graphically examined whether trends in outcomes during the years before Medicaid expansion were parallel in expansion and non-expansion states. States varied in when they expanded Medicaid, so we graphed the trends for each outcome using an “event study” approach that re-scales time to be relative to the year-quarter of Medicaid expansion (Clarke & Tapia-Schythe, 2021). The statistical significance of the pre-policy difference in outcomes for those exposed vs. not exposed to the Medicaid expansion provided a diagnostic of parallel pre-event outcome trends, and the after-policy difference indicated dynamic treatment effects (i.e., whether the policy effects grew or faded over time).

#### Subgroup Analysis

To examine whether the Medicaid expansion had heterogeneous effects, we conducted subgroup analyses by racial/ethnic group. We examined subgroup effects by survey language in supplemental analyses, because prior studies document the highest rates of insurance coverage churn for Spanish-speaking PRAMS respondents (Johnston et al., 2021). Finally, we examine nulliparous births. First births may have high rates of adverse outcomes for birthing parent and infant because the birthing parent cannot draw from experience of prior births and (potentially) pregnancies.

#### Insurance Status Changes

To interpret our findings, we estimate a separate model with the same exposure and control variables but with the outcome as having any prenatal insurance at the time of the first prenatal visit.

## Results

### Main Analysis

Sample characteristics were similar in states that expanded Medicaid and those that did not (Table 1) (Cohen, 1988). All characteristics were included as covariates in the regression analysis to adjust for any observed differences.

**Table 1:** Sample descriptive statistics

	Non-Expansion States		Expansion States	
	(*N* = 64,647)		(*N* = 144,157)	
	Mean (SD), %		Mean (SD), %	
**Panel A. Birthing parent characteristics**				
Age (years)				
<25	46%		43%	
25–34	46%		46%	
35+	8%		11%	
Race				
White	49%		40%	
Black	19%		20%	
Hispanic/Latina	19%		23%	
Other	14%		17%	
Education				
Less than high school	23%		23%	
High school	36%		38%	
Some college	29%		28%	
College or more	11%		11%	
Married	48%		43%	
Income				
Less than $10,000	20%		23%	
$10,000-$19,999	34%		34%	
$20,000-$29,999	18%		17%	
$30,000-$39,999	15%		14%	
$40,000-$49,999	12%		11%	
Parity				
Nulliparous	40%		41%	
Primiparous	29%		29%	
Multiparous	32%		30%	
Family size	2.97 (1.48)		2.91 (1.48)	
**Panel B. Birthing parent outcomes**	Mean (SD), %	No. Obs.	Mean (SD), %	No. Obs.
Month of 1st prenatal visit	2.26 (1.05)	64,201	2.21 (1.11)	143,112
Postpartum visit check-up ^a^	86%	36,042	86%	99,795
Gestational diabetes	11%	64,643	12%	144,150
Hypertensive disorders of pregnancy	8%	64,156	9%	143,492
Smoke last 3 months of pregnancy	18%	63,969	19%	142,439
**Panel C. Infant outcomes**				
Breastfed, ever	80%	62,415	79%	139,731
Breastfed > 1 month^b^	71%	48,720	72%	107,694
Preterm birth	21%	64,594	20%	143,766
Low birth weight	26%	64,647	26%	144,157
Very low birth weight	7%	64,647	4%	144,157

Note: Includes PRAMS survey waves from 2004 to 2017 ^a^ Postpartum visit check-up question was available in Standard Questionnaire 2004–2011 and Core Questionnaire 2012–2017. ^b^ Breastfeeding duration computed only for those that ever breastfed

In the primary analysis, we were unable to reject the null hypothesis of no association between Medicaid expansion with any of the birthing parents’ outcomes, including the timing of the first prenatal visit, gestational diabetes, hypertensive disorders, smoking status, and postpartum check-up visit (Table 2). In most cases, the relatively precise estimates (narrow 95% confidence intervals) rule out any large changes in outcomes. Likewise, we found no association between Medicaid expansion and infant breastfeeding, gestational age, and birth weight.

**Table 2:** Estimated impact of state Medicaid expansion on birthing parent and infant outcomes, by birthing parent race/ethnicity

	Coefficient (95% Confidence Interval)			
	Full Sample	White	Black	Hispanic
**Panel A. Birthing parent outcomes**				
*Health utilization*				
Month of 1st prenatal visit	-0.019	0.011	-0.030	-0.039
	(-0.083–0.045)	(-0.061–0.083)	(-0.099–0.040)	(-0.12–0.039)
Postpartum visit check-up	-0.0077	-0.013	0.021	-0.012
	(-0.020–0.0046)	(-0.028–0.0014)	(-0.0017–0.043)	(-0.037–0.014)
*Health outcomes*				
Gestational diabetes	-0.0020	-0.0085	-0.0045	-0.011
	(-0.018–0.014)	(-0.022–0.0052)	(-0.027–0.018)	(-0.032–0.011)
Hypertensive disorders of pregnancy	-0.00066	0.0032	-0.034	0.011
	(-0.034–0.033)	(-0.018–0.025)	(-0.13–0.061)	(-0.013–0.035)
Smoked last 3 months of pregnancy	-0.0054	-0.0088	-0.011	-0.0035
	(-0.014–0.0034)	(-0.025–0.0073)	(-0.029–0.0078)	(-0.016–0.0089)
**Panel B. Infant outcomes**				
Ever breastfed	0.011	0.020*	-0.0081	0.011
	(-0.013–0.034)	(0.0015–0.039)	(-0.055–0.039)	(-0.0040–0.027)
Breastfed > 1 month	0.0060	-0.0050	0.024	0.027*
	(-0.011–0.023)	(-0.023–0.013)	(-0.0081–0.055)	(0.0034–0.052)
Preterm birth	0.044	0.036	0.048	0.053
	(-0.026–0.11)	(-0.011–0.082)	(-0.065–0.16)	(-0.029–0.13)
Low birth weight	0.050	0.030	0.082	0.043
	(-0.048–0.15)	(-0.041–0.10)	(-0.064–0.23)	(-0.063–0.15)
Very low birth weight	0.033	0.02	0.055	0.032
	(-0.012–0.077)	(-0.0032–0.042)	(-0.032–0.14)	(-0.018–0.082)

Note: Includes PRAMS survey waves from 2004–2017. Sample limited to households with less than $50,000 in income. Each regression model controls for birthing parent’s age, race/ethnicity, education, marital status, household size, income category (under $50K), and parity. Regressions also include state-level covariates and year and state fixed effects. For stratified analysis by race, we did not examine the ‘other’ category separately because the group was too heterogenous

* *p*-value < 0.05

### Subgroup Analyses

When we conducted subgroup analyses by birthing parents’ race, we found that White birthing parents had a 2.3 percentage-point increase in breastfeeding initiation rates after 2014 in Medicaid expansion states. Hispanic birthing parents were 2.6% points more likely to breastfeed for longer than one month after 2014 in Medicaid expansion states. The magnitude of these effect sizes were small, and the coefficients were not statistically significant after accounting for multiple hypothesis testing. We also found no association between Medicaid expansion and any of the outcomes in subgroup analyses by survey language (i.e., English or Spanish, Supplemental Table S3) or for first-time birthing parents (Supplemental Table S4).

### Difference-in-Differences Assumptions

To evaluate the credibility of the difference-in-differences assumptions, we examined differences in the trends in birthing parent and infant outcomes across the two groups of states prior to state Medicaid expansion (Supplement Figure S1 and S2). Results suggest noisy but similar trends prior to Medicaid expansion.

### Insurance Status Changes

We assessed whether the Medicaid expansion changed the likelihood that the birthing parent had any insurance at the first prenatal visit (Supplemental Table S5). While the magnitude of the estimate suggests that there was an increase in the likelihood of having insurance, the estimate was not statistically significant. Thus, the Medicaid expansion had a limited impact on insurance coverage during pregnancy in this sample.

## Discussion

This study examined the relationship between Medicaid expansion and a variety of birthing parent and infant outcomes related to services required under the ACA. Using a large sample covering 23 states, we found no effect of the Medicaid expansion on low-income people regarding the timing of the first prenatal visit, gestational diabetes, hypertensive disorders, smoking status late in pregnancy, and postpartum check-up visit, or on pre-term birth and low birth weight among infants. We found limited effects for breastfeeding practices for White and Hispanic birthing parents, although these results were modest and tentative given the large number of statistical tests conducted.

The largely null results are consistent with previous literature on the impact of Medicaid expansion for pregnant people (Bellerose et al., 2022; Clapp et al., 2019; Daw et al., 2021; Margerison et al., 2021). Given that pregnant people had relatively high insurance coverage even before Medicaid expansion, and cost-sharing in Medicaid has always been low, our results suggest that other barriers to care beyond insurance and cost-sharing are essential to consider. For example, the services associated with several of the birthing parent outcomes require additional appointments, and the birthing parent may face challenges in attending visits in the absence of paid leave in most states or if there are difficulties finding childcare for other children. There are also likely healthcare-related access barriers such as the difficulty of getting Medicaid appointments, limited providers who accept Medicaid, and a lack of respectful maternity care (Allen et al., 2017). These barriers are not easily overcome, even if insurance coverage eliminates the direct costs and providers give necessary referrals. Further, the Medicaid expansion occurred in 2014, so our post-expansion observations were limited to the first three years, during which prior studies document increased wait times for appointments. Longer-term studies might reveal changes in these outcomes as appointment supply and demand equilibrate (Miller & Wherry, 2017).

Our analysis has several notable strengths. The use of PRAMS data linked with birth certificate data allowed us to examine a variety of parental and child outcomes for birthing parent and child dyads. We also adjusted for many more state-level variables that may confound the relationship between Medicaid expansion and outcomes of interest. We undertook several sensitivity analyses to ensure the robustness of the findings. Nonetheless, the use of PRAMS has some sample limitations. Specifically, we included 23 states from PRAMS where data were available. In this sample, the Medicaid expansion had limited impact on insurance at the first prenatal visit, but the results may vary if we had data from all 50 states. Our sample excluded the two largest states, California and Texas, with different Medicaid policies and large populations of low-income parents. Further, there may be some systematic selection due to income. Our analytic sample excluded 5.85% of respondents who did not have income data but had complete data otherwise. Complete case analysis is not thought to result in bias at such low levels of missingness (Allison, 2009; Bennett, 2001; Dong & Peng, 2013; Langkamp et al., 2010). Finally, we do not account for systematic non-response to the follow-up survey, which might limit external generalizability.

## Conclusion

While better access to more complete insurance may impact some pregnant people, especially those with pre-existing conditions (Breathett et al., 2018), it may not address more fundamental structural barriers that affect many low-income people. Prior research suggests that more upstream social safety net programs such as food assistance and paid family leave improve birthing parent and infant outcomes (Almond et al., 2011; Hamad et al., 2019). Income support programs such as the EITC have also been shown to improve perinatal health outcomes, suggesting the potential for poverty alleviation policies to reduce income disparities while also improving health (Hamad & Rehkopf, 2015; Hoynes et al., 2015). Considering that low-income people face multiple barriers and have worse health than higher income individuals, future research should identify polices and interventions that address root causes of existing disparities. Such interventions are necessary in addition to health insurance to ensure that children and their families start out as healthy as possible to promote health and wellbeing throughout life.

### Electronic Supplementary Material

Below is the link to the electronic supplementary material.

**Supplementary Appendix: Supplemental Table S1**. Number of Respondents by State and Year. **Supplemental Table S2**. Medicaid and CHIP Income Eligibility Limits for Pregnant People relative to Federal Poverty Line in Study, 2011–2017. **Supplemental Table S3**. Estimated Impact of State Medicaid Expansion on Birthing Parent and Infant Outcomes, by Survey Language. **Supplemental Table S4**. Estimated Impact of State Medicaid Expansion on Birthing Parent and Infant Outcomes, Comparing Full Sample to Nulliparous Birthing Parents. **Supplemental Table S5**. Estimated Impact of State Medicaid Expansion on Having Any Insurance at First Prenatal Visit. **Supplemental Figure S1**. Event-Study Estimates of Difference by Treatment Group and Parallel Pre-ACA Trends for Birthing Parent Outcomes. **Supplemental Figure S2**: Event-Study Estimates of Difference by Treatment Group and Parallel Pre-ACA Trends for Infant Outcomes

## Data Availability

The data that support the findings of this study are available from PRAMS and researchers may submit a proposal to PRAMS for access to the data.
