# Supplementary material for: Medicaid Expansion and Perinatal Health Outcomes: A Quasi-Experimental Study
Source: Matern Child Health J. 2024 Jan 20;28(5):959–68. doi: 10.1007/s10995-023-03879-y (PMC11001670; doi:10.1007/s10995-023-03879-y)
Supplement: Supplementary file 1 — Supplementary Appendix: Supplemental Table S1. Number of Respondents by State and Year. Supplemental Table S2. Medicaid and CHIP Income Eligibility Limits for Pregnant People relative to Federal Poverty Line in Study, 2011–2017. Supplemental Table S3. Estimated Impact of State Medicaid Expansion on Birthing Parent and Infant Outcomes, by Survey Language. Supplemental Table S4. Estimated Impact of State Medicaid Expansion on Birthing Parent and Infant Outcomes, Comparing Full Sample to Nulliparous Birthing Parents. Supplemental Table S5. Estimated Impact of State Medicaid Expansion on Having Any Insurance at First Prenatal Visit. Supplemental Figure S1. Event-Study Estimates of Difference by Treatment Group and Parallel Pre-ACA Trends for Birthing Parent Outcomes. Supplemental Figure S2: Event-Study Estimates of Difference by Treatment Group and Parallel Pre-ACA Trends for Infant Outcomes [file 10995_2023_3879_MOESM1_ESM.docx]

**Supplementary Appendix**

Supplement to: Modrek S, Collin DF, Hamad R, White JS. “Medicaid Expansion and Perinatal Health Outcomes: A Quasi-Experimental Study”

Table of Contents

[Supplemental Table S1. Number of Respondents by State and Year 2](#_Toc149644174)

[Supplemental Table S2. Medicaid and CHIP Income Eligibility Limits for Pregnant People relative to Federal Poverty Line in Study, 2011-2017 3](#_Toc149644175)

[Supplemental Table S3. Estimated Impact of State Medicaid Expansion on Birthing Parent and Infant Outcomes, by Survey Language 4](#_Toc149644176)

[Supplemental Table S4. Estimated Impact of State Medicaid Expansion on Birthing Parent and Infant Outcomes, Comparing Full Sample to Nulliparous Birthing Parents 5](#_Toc149644177)

[Supplemental Table S5. Estimated Impact of State Medicaid Expansion on Having Any Insurance at First Prenatal Visit 6](#_Toc149644178)

[Supplemental Figure S1. Event-Study Estimates of Difference by Treatment Group and Parallel Pre-ACA Trends for Birthing Parent Outcomes 7](#_Toc149644179)

[Supplemental Figure S2: Event-Study Estimates of Difference by Treatment Group and Parallel Pre-ACA Trends for Infant Outcomes 8](#_Toc149644180)

# **Supplemental Table S1.** Number of Respondents by State and Year

|  | **Year** | | | | | | | | | | | | | | |
| --- | --- | --- | --- | --- | --- | --- | --- | --- | --- | --- | --- | --- | --- | --- | --- |
|  | 2004 | 2005 | 2006 | 2007 | 2008 | 2009 | 2010 | 2011 | 2012 | 2013 | 2014 | 2015 | 2016 | 2017 | Total |
| **Expansion States** |  |  |  |  |  |  |  |  |  |  |  |  |  |  |  |
| Alaska | 682 | 689 | 654 | 862 | 732 | 667 | 604 | 0 | 432 | 647 | 537 | 614 | 596 | 530 | 8,246 |
| Arkansas | 1,407 | 1,506 | 1,396 | 1,349 | 1,175 | 842 | 1,141 | 995 | 592 | 767 | 0 | 632 | 606 | 0 | 12,408 |
| Colorado | 881 | 894 | 877 | 1,106 | 1,074 | 1,082 | 1,087 | 1,033 | 586 | 778 | 0 | 889 | 688 | 498 | 11,473 |
| Delaware | 0 | 0 | 0 | 354 | 691 | 618 | 590 | 577 | 536 | 573 | 522 | 499 | 431 | 421 | 5,812 |
| Hawaii | 963 | 760 | 723 | 937 | 957 | 898 | 923 | 921 | 770 | 816 | 713 | 691 | 172 | 0 | 10,244 |
| Illinois | 826 | 713 | 715 | 855 | 778 | 812 | 741 | 716 | 492 | 588 | 658 | 713 | 564 | 490 | 9,661 |
| Massachusetts | 0 | 0 | 0 | 762 | 748 | 703 | 786 | 777 | 735 | 690 | 764 | 633 | 555 | 683 | 7,836 |
| Maryland | 501 | 423 | 479 | 642 | 675 | 629 | 646 | 599 | 400 | 519 | 595 | 543 | 489 | 430 | 7,570 |
| Michigan | 568 | 568 | 556 | 876 | 978 | 1,072 | 847 | 1,011 | 1,222 | 1,270 | 0 | 897 | 951 | 1,032 | 11,848 |
| New Jersey | 938 | 948 | 746 | 705 | 671 | 651 | 642 | 615 | 443 | 391 | 573 | 498 | 550 | 479 | 8,850 |
| New York^a^ | 755 | 969 | 953 | 1,329 | 0 | 0 | 1,244 | 1,243 | 0 | 1,227 | 994 | 1,136 | 1,077 | 880 | 11,807 |
| Oregon | 1,066 | 1,034 | 1,012 | 1,149 | 1,061 | 1,082 | 1,195 | 1,162 | 458 | 891 | 0 | 877 | 0 | 0 | 10,987 |
| Pennsylvania | 0 | 0 | 0 | 400 | 556 | 538 | 495 | 503 | 327 | 473 | 534 | 494 | 411 | 538 | 5,269 |
| Rhode Island | 623 | 555 | 541 | 694 | 662 | 648 | 603 | 616 | 557 | 534 | 596 | 0 | 552 | 491 | 7,672 |
| Washington | 817 | 743 | 775 | 849 | 966 | 1,021 | 947 | 736 | 495 | 547 | 595 | 627 | 589 | 563 | 10,270 |
| West Virginia | 565 | 1,027 | 1,115 | 1,258 | 1,183 | 1,092 | 971 | 1,043 | 694 | 862 | 774 | 722 | 459 | 365 | 12,130 |
| **Non-Expansion States** |  |  |  |  |  |  |  |  |  |  |  |  |  |  |  |
| Georgia | 785 | 918 | 966 | 389 | 543 | 543 | 722 | 916 | 424 | 310 | 0 | 0 | 0 | 488 | 7,004 |
| Maine | 509 | 506 | 525 | 621 | 632 | 603 | 599 | 558 | 361 | 497 | 523 | 407 | 382 | 401 | 7,124 |
| Nebraska | 913 | 976 | 882 | 970 | 963 | 1,089 | 1,115 | 1,057 | 629 | 847 | 834 | 775 | 667 | 0 | 11,717 |
| Oklahoma | 1,070 | 1,099 | 1,133 | 1,379 | 1,271 | 1,352 | 1,208 | 1,248 | 1,031 | 1,052 | 1,059 | 1,178 | 1,236 | 1,037 | 16,353 |
| Utah | 1,086 | 1,025 | 1,062 | 1,079 | 1,072 | 969 | 949 | 862 | 903 | 737 | 813 | 765 | 665 | 703 | 12,690 |
| Wisconsin | 0 | 0 | 0 | 625 | 651 | 597 | 0 | 1,097 | 1,028 | 958 | 975 | 917 | 691 | 808 | 8,347 |
| Wyoming | 0 | 0 | 0 | 571 | 549 | 524 | 608 | 398 | 306 | 325 | 324 | 303 | 321 | 255 | 4,484 |

Note: Data from PRAMS survey waves from 2004-2017. Coverage under the Medicaid expansion became effective January 1, 2014 in all states that have adopted the Medicaid expansion except for the following: Michigan (4/1/2014), Alaska (9/1/2015), and Pennsylvania (1/1/2015).

^a^ Although administered separately, responses for New York State and New York City were aggregated into one group for the purposes of this study.

# **Supplemental Table S2**. Medicaid and CHIP Income Eligibility Limits for Pregnant People relative to Federal Poverty Line in Study, 2011-2017

|  | **Year** | | | | | | | |
| --- | --- | --- | --- | --- | --- | --- | --- | --- |
|  | Jan-11 | Jan-12 | Jan-13 | Jan-14 | Jan-15 | Jan-16 | Jan-17 |  |
| **Expansion States** |  |  |  |  |  |  |  |  |
| Alaska | 1.75 | 1.75 | 1.75 | 2.05 | 2.05 | 2.05 | 2.05 |  |
| Arkansas | 2 | 2 | 2 | 2.14 | 2.14 | 2.14 | 2.14 |  |
| Colorado | 2.5 | 2.5 | 2.5 | 2.65 | 2.65 | 2.65 | 2.65 |  |
| Delaware | 2 | 2 | 2 | 2.14 | 2.17 | 2.17 | 2.17 |  |
| Hawaii | 1.85 | 1.85 | 1.85 | 1.96 | 1.96 | 1.96 | 1.96 |  |
| Illinois | 2 | 2 | 2 | 2.13 | 2.13 | 2.13 | 2.13 |  |
| Maryland | 2.5 | 2.5 | 2.5 | 2.64 | 2.64 | 2.64 | 2.64 |  |
| Massachusetts | 2 | 2 | 2 | 2.05 | 2.05 | 2.05 | 2.05 |  |
| Michigan | 1.85 | 1.85 | 1.85 | 2 | 2 | 2 | 2 |  |
| New Jersey | 2 | 2 | 2 | 2.05 | 2.05 | 2.05 | 2.05 |  |
| New York | 2 | 2 | 2 | 2.23 | 2.23 | 2.23 | 2.23 |  |
| Oregon | 1.85 | 1.85 | 1.85 | 1.9 | 1.9 | 1.9 | 1.9 |  |
| Pennsylvania | 1.85 | 1.85 | 1.85 | 2.2 | 2.2 | 2.2 | 2.2 |  |
| Rhode Island | 2.5 | 2.5 | 2.5 | 2.58 | 2.58 | 2.58 | 2.58 |  |
| Washington | 1.85 | 1.85 | 1.85 | 1.98 | 1.98 | 1.98 | 1.98 |  |
| West Virginia | 1.5 | 1.5 | 1.5 | 1.63 | 1.63 | 1.63 | 1.63 |  |
| **Non-Expansion States** |  |  |  |  |  |  |  |  |
| Georgia | 2 | 2 | 2 | 2.25 | 2.25 | 2.25 | 2.25 |  |
| Maine | 2 | 2 | 2 | 2.14 | 2.14 | 2.14 | 2.14 |  |
| Nebraska | 1.85 | 1.85 | 1.85 | 2.02 | 2.02 | 2.02 | 2.02 |  |
| Oklahoma | 1.85 | 1.85 | 1.85 | 1.9 | 1.9 | 1.9 | 2.1 |  |
| Utah | 1.33 | 1.33 | 1.33 | 1.44 | 1.44 | 1.44 | 1.44 |  |
| Wisconsin | 3 | 3 | 3 | 3.06 | 3.06 | 3.06 | 3.06 |  |
| Wyoming | 1.33 | 1.33 | 1.33 | 1.59 | 1.59 | 1.59 | 1.59 |  |
| Federal Limits in Dollars |  |  |  |  |  |  |  |  |
| Federal Poverty Line (Family of 2) | $14,710 | $15,130 | $15,510 | $15,730 | $15,930 | $16,020 | $16,240 |  |
| Federal Poverty Line (Family of 4) | $22,350 | $23,050 | $23,550 | $23,850 | $24,250 | $24,300 | $24,600 |  |
| Expansion States Mean Eligibility Limit (Fam. of 2) | $29,420 | $30,260 | $31,020 | $33,751 | $34,210 | $34,403 | $34,875 |  |
| Non-Expansion States Mean Eligibility Limit (Fam. of 2) | $28,075 | $28,877 | $29,602 | $32,359 | $32,770 | $32,955 | $33,872 |  |
| Expansion States Mean Eligibility Limit (Fam. of 4) | $44,700 | $46,100 | $47,100 | $51,173 | $52,077 | $52,184 | $52,829 |  |
| Non-Expansion States Mean Eligibility Limit (Fam. of 4) | $42,657 | $43,993 | $44,947 | $49,063 | $49,886 | $49,989 | $51,309 |  |

Source:

<https://www.kff.org/medicaid/state-indicator/medicaid-and-chip-income-eligibility-limits-for-pregnant-women/?currentTimeframe=0&sortModel=%7B%22colId%22:%22Location%22,%22sort%22:%22asc%22%7D>

<https://aspe.hhs.gov/topics/poverty-economic-mobility/poverty-guidelines/prior-hhs-poverty-guidelines-federal-register-references>

# **Supplemental Table S3.** Estimated Impact of State Medicaid Expansion on Birthing Parent and Infant Outcomes, by Survey Language

|  | Coefficient (95% Confidence Interval) | | |
| --- | --- | --- | --- |
|  | Full Sample | English Survey | Spanish Survey |
| **Panel A. Birthing Parent outcomes** |  |  |  |
| *Health utilization* |  |  |  |
| Month of 1st prenatal visit | -0.019 | -0.017 | -0.0093 |
|  | (-0.083 - 0.045) | (-0.083 - 0.049) | (-0.14 - 0.12) |
| Postpartum visit check-up | -0.0077 | -0.0071 | 0.0042 |
|  | (-0.020 - 0.0046) | (-0.020 - 0.0058) | (-0.023 - 0.032) |
| *Health outcomes* |  |  |  |
| Gestational diabetes | -0.0020 | 0.00082 | -0.018 |
|  | (-0.018 - 0.014) | (-0.014 - 0.016) | (-0.049 - 0.013) |
| Hypertensive disorders of pregnancy | -0.00066 | -0.0016 | 0.012 |
|  | (-0.034 - 0.033) | (-0.037 - 0.034) | (-0.012 - 0.037) |
| Smoked last 3 months of pregnancy | -0.0054 | -0.0081 | -0.0050 |
|  | (-0.014 - 0.0034) | (-0.019 - 0.0028) | (-0.012 - 0.0017) |
|  |  |  |  |
|  |  |  |  |
| **Panel B. Infant outcomes** |  |  |  |
| Ever breastfed | 0.011 | 0.0087 | 0.0014 |
|  | (-0.013 - 0.034) | (-0.016 - 0.033) | (-0.024 - 0.027) |
| Breastfed > 1 month | 0.0060 | 0.0048 | 0.023 |
|  | (-0.011 - 0.023) | (-0.011 - 0.020) | (-0.0095 - 0.056) |
| Preterm birth | 0.044 | 0.044 | 0.024 |
|  | (-0.026 - 0.11) | (-0.025 - 0.11) | (-0.065 - 0.11) |
| Low birthweight | 0.050 | 0.048 | 0.021 |
|  | (-0.048 - 0.15) | (-0.048 - 0.14) | (-0.091 - 0.13) |
| Very low birthweight | 0.033 | 0.032 | 0.024 |
|  | (-0.012 - 0.077) | (-0.012 - 0.077) | (-0.022 - 0.069) |

Note: Includes PRAMS survey waves from 2004-2017 unless otherwise noted. Sample limited to household with incomes less than $50,000. Each regression model controls for birthing parent’s age, race/ethnicity, education, marital status, income, household size and parity. Regressions also include state-level covariates, and year and state fixed effects.

* p-value < 0.05

# **Supplemental Table S4.** Estimated Impact of State Medicaid Expansion on Birthing Parent and Infant Outcomes, Comparing Full Sample to Nulliparous Birthing Parents

|  | Coefficient (95% Confidence Interval) | |
| --- | --- | --- |
|  | Full Sample | Nulliparous |
| **Panel A. Birthing Parent** |  |  |
| *Health utilization* |  |  |
| Month of 1st prenatal visit | -0.019 | -0.030 |
|  | (-0.083 - 0.045) | (-0.098 - 0.038) |
| Postpartum visit check-up | -0.0077 | -0.0037 |
|  | (-0.020 - 0.0046) | (-0.023 - 0.015) |
| *Health outcomes* |  |  |
| Gestational diabetes | -0.0020 | -0.0024 |
|  | (-0.018 - 0.014) | (-0.013 - 0.0087) |
| Hypertensive disorders of pregnancy | -0.00066 | 0.0046 |
|  | (-0.034 - 0.033) | (-0.028 - 0.038) |
| Smoked last 3 months of pregnancy | -0.0054 | -0.0028 |
|  | (-0.014 - 0.0034) | (-0.022 - 0.016) |
|  |  |  |
| **Panel B. Infant outcomes** |  |  |
| Ever breastfed | 0.011 | 0.0098 |
|  | (-0.013 - 0.034) | (-0.017 - 0.036) |
| Breastfed > 1 month | 0.0060 | 0.0077 |
|  | (-0.011 - 0.023) | (-0.013 - 0.028) |
| Preterm birth | 0.044 | 0.048 |
|  | (-0.026 - 0.11) | (-0.027 - 0.12) |
| Low birthweight | 0.050 | 0.051 |
|  | (-0.048 - 0.15) | (-0.053 - 0.15) |
| Very low birthweight | 0.033 | 0.043 |
|  | (-0.012 - 0.077) | (-0.0098 - 0.095) |

Note: Includes PRAMS survey waves from 2004-2017 unless otherwise noted. Sample limited to household with incomes less than $50,000. Each regression model controls for birthing parent’s age, race/ethnicity, education, marital status, income, household size and parity. Regressions also include state-level covariates, and year and state fixed effects.

* p-value < 0.05

# **Supplemental Table S5.** Estimated Impact of State Medicaid Expansion on Having Any Insurance at First Prenatal Visit

|  | Prenatal Care Paid for by Any Insurance | |
| --- | --- | --- |
| Variable | Coef. | 95% CI |
| Post-Medicaid Expansion | 0.025 | (-0.047 - 0.096) |
| Age (Ref. <25) |  |  |
| 25-34 | 0.081** | (0.052 - 0.11) |
| 35+ | 0.13** | (0.097 - 0.17) |
| Race (Ref. White) |  |  |
| Black | 0.012 | (-0.046 - 0.070) |
| Hispanic/Latina | 0.22* | (0.053 - 0.38) |
| Other | 0.074** | (0.020 - 0.13) |
| Education (Ref. Less than HS) |  |  |
| HS | -0.084** | (-0.13 - -0.033) |
| Some college | -0.074* | (-0.13 - -0.015) |
| College + | 0.031 | (-0.036 - 0.098) |
| Married | 0.14** | (0.12 - 0.17) |
| Income (Ref. $10,000) |  |  |
| $10,000-$19,999 | 0.066** | (0.047 - 0.084) |
| $20,000-$29,999 | 0.16** | (0.12 - 0.19) |
| $30,000-$39,999 | 0.30** | (0.27 - 0.34) |
| $40,000-$49,999 | 0.44** | (0.41 - 0.48) |
| Family size | -0.0097* | (-0.018 - -0.0012) |
| Parity (Ref. Nulliparous) |  |  |
| Primiparous | -0.093** | (-0.11 - -0.076) |
| Multiparous | -0.16** | (-0.18 - -0.13) |
| Constant | 2.33 | (-1.66 - 6.33) |
| Observations | 202,533 | |
| R-squared | 0.137 | |

Note: Model includes state-level time-varying covariates, and year and state fixed effects. Standard errors were clustered by state. Robust confidence intervals in parentheses.

* p-value < 0.05 ** p-value <0.01

# **Supplemental Figure S1.** Event-Study Estimates of Difference by Treatment Group and Parallel Pre-ACA Trends for Birthing Parent Outcomes

**
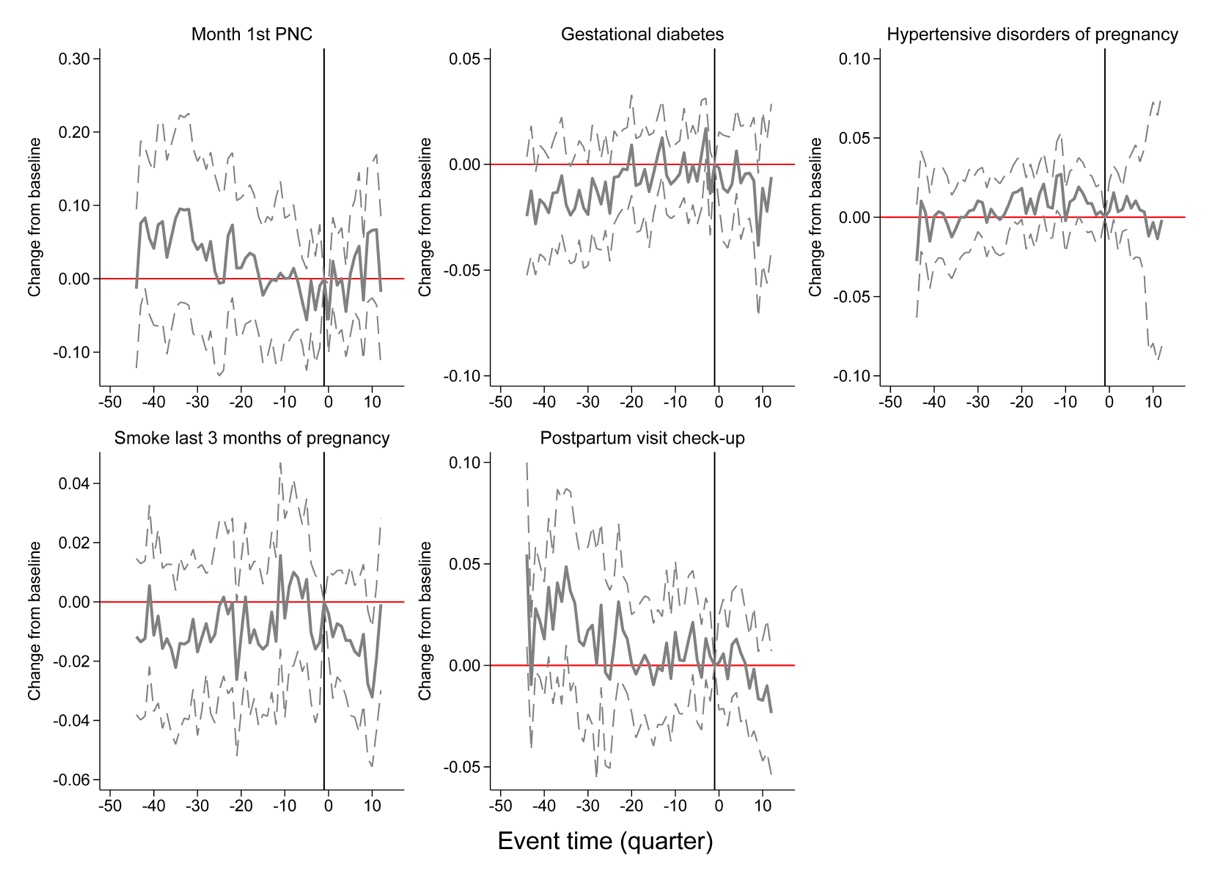
**

Note: Means and 95% confidence intervals of difference between treatment and control groups compared to differences between treatment and control groups in the first quarter of 2014 (the omitted category that is set to 0).

# Supplemental Figure S2: Event-Study Estimates of Difference by Treatment Group and Parallel Pre-ACA Trends for Infant Outcomes

**
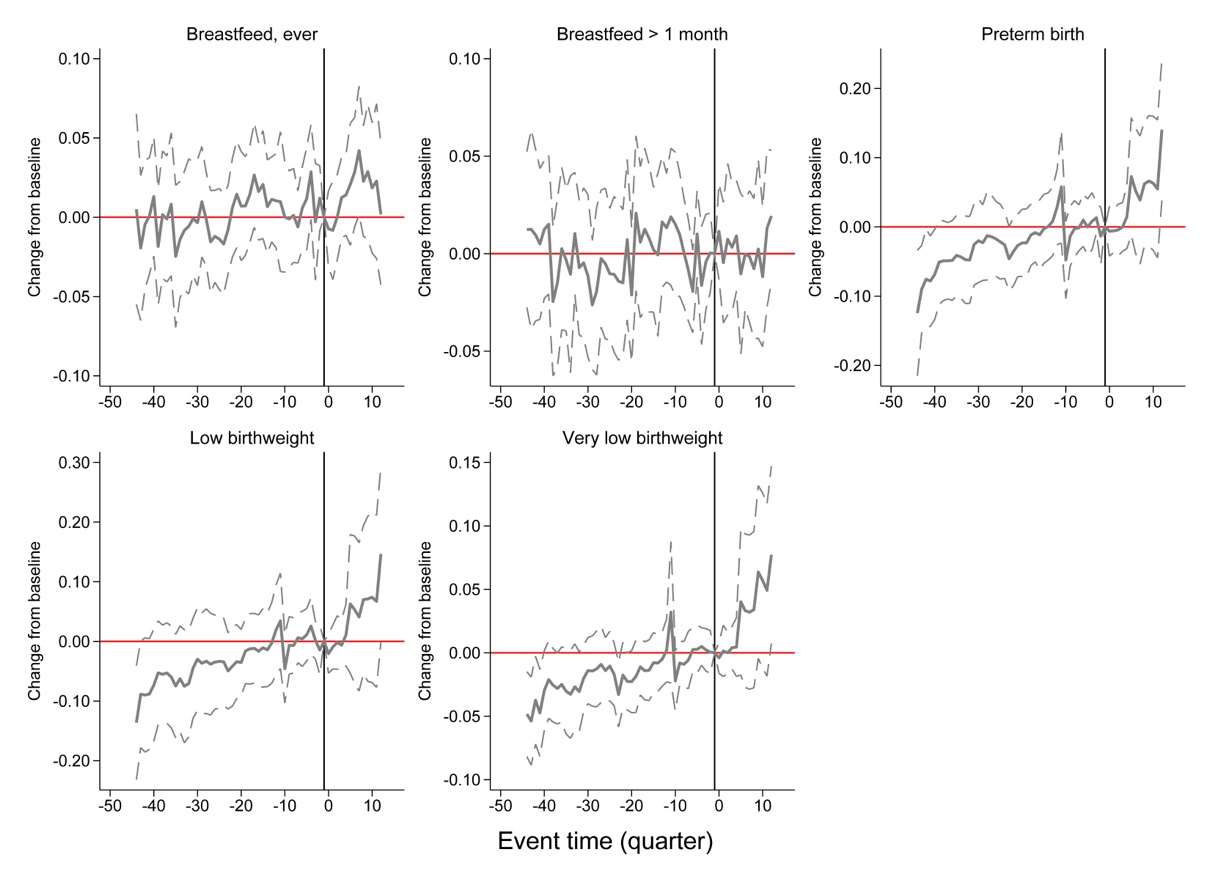
**

Note: Means and 95% confidence intervals of difference between treatment and control groups compared to differences between treatment and control groups in the first quarter of 2014 (the omitted category that is set to 0).
